# DeepASD: a deep adversarial-regularized graph learning method for ASD diagnosis with multimodal data

**DOI:** 10.1038/s41398-024-02972-2

**Published:** 2024-09-14

**Authors:** Wanyi Chen, Jianjun Yang, Zhongquan Sun, Xiang Zhang, Guangyu Tao, Yuan Ding, Jingjun Gu, Jiajun Bu, Haishuai Wang

**Affiliations:** 1 Department of Hepatobiliary and Pancreatic Surgery, The Second Affiliated Hospital, Zhejiang University School of Medicine, Hangzhou, Zhejiang China; 2 Zhejiang Provincial Key Laboratory of Service Robot, College of Computer Science, Zhejiang University, Hangzhou, China; 3 Department of General Practice, Shandong Provincial Third Hospital, Shandong University, Jinan, Shandong China; 4 Department of Computer Science, University of North Carolina at Charlotte, Charlotte, NC USA; 5 Department of Radiology, Shanghai Chest Hospital, Shanghai Jiao Tong University School of Medicine, Shanghai, China; 6 Pujian Technology, Hangzhou, Zhejiang China; 7 Shanghai Artificial Intelligence Laboratory, Shanghai, China

**Keywords:** Autism spectrum disorders, Predictive markers

## Abstract

Autism Spectrum Disorder (ASD) is a prevalent neurological condition with multiple co-occurring comorbidities that seriously affect mental health. Precisely diagnosis of ASD is crucial to intervention and rehabilitation. A single modality may not fully reflect the complex mechanisms underlying ASD, and combining multiple modalities enables a more comprehensive understanding. Here, we propose, DeepASD, an end-to-end trainable regularized graph learning method for ASD prediction, which incorporates heterogeneous multimodal data and latent inter-patient relationships to better understand the pathogenesis of ASD. DeepASD first learns cross-modal feature representations through a multimodal adversarial-regularized encoder, and then constructs adaptive patient similarity networks by leveraging the representations of each modality. DeepASD exploits inter-patient relationships to boost the ASD diagnosis that is implemented by a classifier compositing of graph neural networks. We apply DeepASD to the benchmarking Autism Brain Imaging Data Exchange (ABIDE) data with four modalities. Experimental results show that the proposed DeepASD outperforms eight state-of-the-art baselines on the benchmarking ABIDE data, showing an improvement of 13.25% in accuracy, 7.69% in AUC-ROC, and 17.10% in specificity. DeepASD holds promise for a more comprehensive insight of the complex mechanisms of ASD, leading to improved diagnosis performance.

## Introduction

Autism spectrum disorder (ASD) is one of the most common neurodevelopmental disorders, mainly characterized by qualitative impairments in social functioning [1–6]. With 1 in 36 children suffering from ASD [7], ASD severely affects their quality of life such as social communication and interaction. Precise and timely diagnosis of ASD can trigger earlier intervention and bring positive long-term outcomes in communication skills, verbal and cognitive abilities, etc. [2] The mainstream of ASD clinical diagnosis depends on traditional behavioral criteria [8], which often results in delayed diagnosis or misdiagnosis due to being time-consuming and technically demanding. Beyond behavioral evaluations, a number of MRI-based computational approaches attempt to construct functional connectivity (FC) maps to learn abnormal connectivity between brain regions, and then employ deep neural networks to differentiate ASD and typical controls (TCs).

However, deep learning-based automatic diagnosis techniques have, in most cases, focused on single-modal MRI data. Compared to single brain atlas fMRI, different brain atlases [9–11] and heterogeneous medical data from different modalities [12–14] can provide complementary information to each other. Nevertheless, there have not been superb multimodal-based results obtained in the clinical evaluation of ASD. Due to the diversity of the underlying distribution and complicated associations across modalities [15], leveraging a variety of types of data to encode more detailed and comprehensive information for accurate ASD diagnosis is challenging. Pioneer methods have imposed a graph convolution network (GCN) [16–18] to aggregate knowledge and learn a patient similarity network. Although it is documented that patient similarities can provide complementary knowledge for ASD diagnosis [19], existing approaches have mainly emphasized the intra-modal knowledge from a single modality for patient similarity network construction while rarely considering both intra-modal and cross-modal information to enhance patient alignments.

To address these challenges, we propose an end-to-end adaptive graph learning framework, named DeepASD, to identify ASD by exploiting multimodal representations of functional Magnetic Resonance Imaging (fMRI) and non-imaging phenotypic data, aiming to construct a unified graph to represent knowledge and patient similarity for classification. Specifically, the distributions of different modalities are first aligned using the most informative modality (i.e., fMRI) by training an adversarial-regularized encoder (i.e., a feature transformer and a discriminator) for each modality. The intra-modal knowledge within each modality is then represented as a graph to reveal the connections among patients reflected within each modality. DeepASD further aggregates cross-modal knowledge to align the different modalities for patient representations, and adopts a graph convolution network to identify ASD diagnoses. We evaluate DeepASD on two datasets from the Autism Brain Imaging Data Exchange (ABIDE). DeepASD exhibited improved diagnostic ability for ASD compared to alternative approaches. The model achieved the following performance metrics, presented in mean ± standard deviation format, on the ABIDE A dataset: accuracy (ACC) of 87.38 ± 2.87$\%$, area under curve (AUC) of 92.76 ± 4.00$\%$, specificity (SEN) of 88.35 ± 6.83$\%$, and sensitivity (SPE) of 86.51 ± 8.41$\%$. Similarly, on the ABIDE B dataset, the results were ACC 88.09 ± 2.92$\%$, AUC 93.59 ± 2.45$\%$, SEN 87.58 ± 3.68$\%$, and SPE 88.49 ± 4.73$\%$. We visualized the features learned by our multimodal adversarial-regularized encoder, showing that the different modal features still maintain their specificity but are more distinguishable than the original features in the reduced-dimensional space. We also visualized and analyzed the learned patient similarity graphs, revealing that the learned patient similarity networks are highly correlated with age and sex. In addition, we found that the fMRI modality is significantly correlated with ASD diagnoses in the multimodal datasets. The findings are consistent with the demographic disparities recorded in autism diagnoses [20]. Therefore, we conclude that DeepASD is a promising tool for ASD diagnosis by leveraging multimodal clinical data.

## Results

**Fig. 1:**
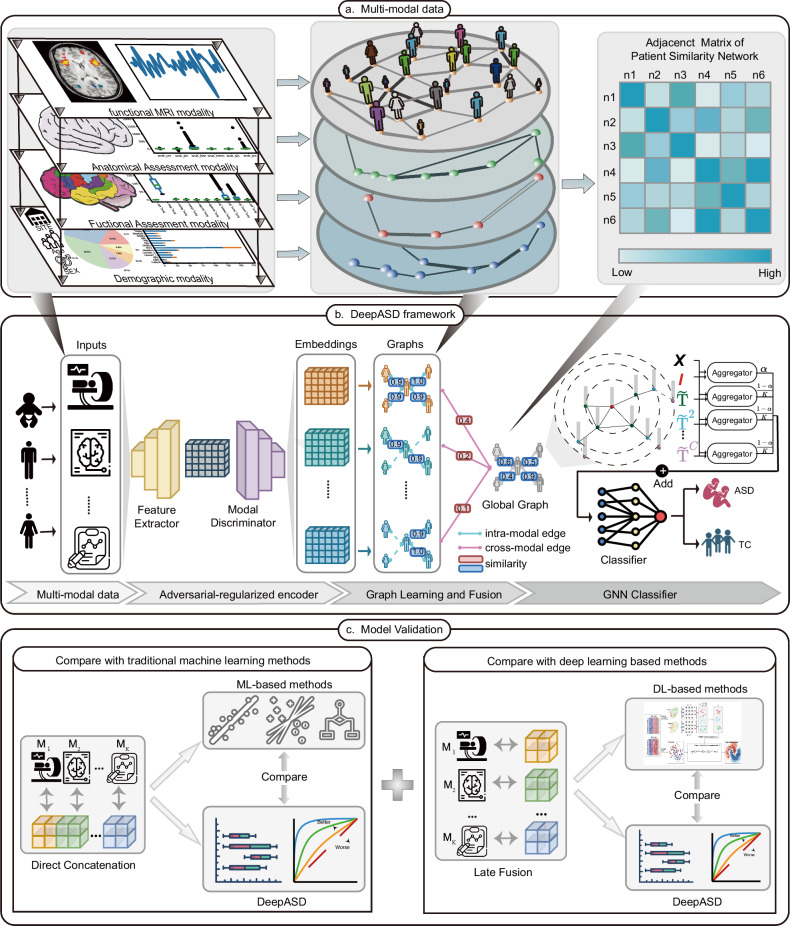
The workflow of DeepASD. **a** Multimodal data for ASD diagnosis. The left panel shows that the multimodal datasets consist of fMRI, automated anatomical quality assessment metrics (ANAT), automated functional quality assessment metrics (FUNC), and demographic information (PHENO). According to the information of the multimodal data, DeepASD constructs a multimodal patient similarity network as shown in the middle panel. Using the weighted fusion method (weights are automatically learned), we obtain a global patient similarity network from the multimodal patient similarity network. The right panel presents the global patient similarity matrix (taking six patients as an example). The darker the color, the more similar of the patient embeddings. **b** The proposed DeepASD framework. DeepASD first adopts an adversarial-regularized encoder module to align the embeddings from different modalities, thereby the learned embeddings will be aligned into the same latent space. We then construct a patient similarity graph for each modality, where each graph node denotes one patient and the edges denote the inter-patient connections. After that, we fuse the multiple constructed modality-specific graphs into a global graph that represents the patient similarity globally. Finally, we employ a graph neural network classifier for ASD diagnosis based on the inter-patient global graph and aligned embeddings. **c** Model validation. We compare the proposed DeepASD with six state-of-the-art baselines, including three traditional machine learning-based models and three deep learning-based models, on two benchmarking datasets. The proposed DeepASD outperforms all baselines significantly.

Given the multimodal medical data, DeepASD applies adversarial representation learning to align embeddings of different modalities, and exploits intra-modal correlations, aiming to construct a joint patient similarity network for ASD identification. DeepASD consists of three staged (Fig. 1b; Section “Methods”): an adversarial-regularized encoder, graph learning and fusion, and a GNN classifier. We first develop a multimodal adversarial-regularized encoder to cope with the feature heterogeneity of each modality and reduce the distributional divergence. Through the graph learning module, we then construct the patient similarity network for each modality independently and generate a global adjacency matrix by leveraging knowledge from all modalities. Finally, DeepASD identifies ASD by feeding the learned global adjacency matrix and aligned feature embeddings into a graph neural network (GNN) classifier.

### Datasets

DeepASD was evaluated on the benchmarking dataset of ABIDE [21], which consists of fMRI images and corresponding phenotypic observations. Specifically, there are two multimodal datasets in the ABIDE data: (i) ABIDE A, which contains four modalities, i.e., demographic information, automated anatomical quality assessment metrics, automated functional quality assessment metrics, and fMRI connection networks. The dataset includes 871 subjects (468 TC and 403 ASD subjects). We follow (Parisot, 2017) [19] for the dataset settings and preprocessing. (ii) ABIDE B, encompassing 949 subjects, adheres to the settings outlined by (Wang, 2020) [10]. This cohort includes 419 individuals diagnosed with ASD and 530 TC subjects. The dataset integrates three brain atlases (CC200, AAL - Automated Anatomical Labeling, and DOS - Dosenbach160) along with demographic information to create a multimodal dataset comprising four modalities. The reason why the dataset was split into ABIDE A and ABIDE B is because of different purpose and specific objectives for analysis [10, 19]. Specifically, ABIDE A divides phenotypic information into detailed demographic information, automated anatomical quality assessment metrics, and automated functional quality assessment metrics. This subdivision facilitates a comprehensive investigation of the interplay between various phenotypic information and a single atlas in fMRI diagnostics. On the other hand, ABIDE B categorizes the fMRI modality based on different atlases (i.e., CC200, AAL, and DOS) and integrates it using demographic information, aiming to explore the diagnostic implications of combining multiple fMRI types with demographic information. For a fair comparison, all data are preprocessed using the Configurable Pipeline for the Analysis of Connectomes (C-PAC) based on the preprocessed fMRI datasets [22].

### DeepASD enables effective multi-modal data integration and accurate prediction of ASD

The proposed DeepASD model was compared with other shallow machine learning methods and recently reported multimodal deep learning models. To obtain an unbiased estimation and more robust performance for ASD diagnosis, we performed a 10-fold stratified cross-validation strategy on the two datasets. To eliminate the effects of randomness in the experiments, we employed an independent two-sample *t*-test to calculate *P*-value.

**Fig. 2:**
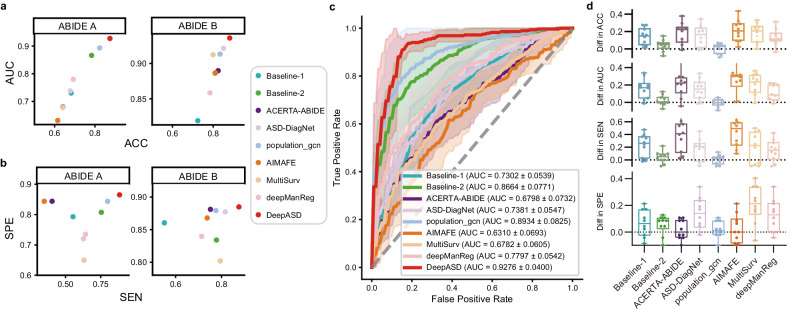
Performance of DeepASD on ABIDE A and ABIDE B. Panels **a** and **b** illustrate the classification performance in a two-dimensional manner on the two datasets. Panel **c** shows the ROC curves of DeepASD compared with state-of-the-art baselines on the ABIDE A dataset. The mean ROC curve and standard deviation of 10-fold cross-validation results are shown as bold lines and shad regions, respectively. Panel **d** reports the performance margin (in terms of ACC, AUC, SEN, and SPE), showing DeepASD outperforms other baselines. Each dot denotes a fold among the 10-fold cross-validation.

Figure 2a shows the AUC and ACC of multiple methods. On the ABIDE A dataset, DeepASD achieved an ACC of 87.38 ± 2.87%, which significantly outperforms all baselines (*P*-vacitelue $<$ 0.05). On ABIDE B, the of our DeepASD is also significantly higher than that of baselines, as shown in Table 1. Additionally, Fig. 2b illustrates that DeepASD outperforms other methods in terms of SEN and SPE on both ABIDE A and ABIDE B datasets. In measuring SEN and SPE, we adhere to a cutoff of 0.5, as per our experimental methodology utilizing deep learning techniques. This choice is made due to our dataset presenting a balanced distribution of positive and negative samples, thus warranting a fixed threshold of 0.5. Moreover, as shown in Fig. 2c, DeepASD also achieves a relatively high area under the receiver operating characteristic (ROC) curve AUC values of 92.76 ± 4.00% on ABIDE A.

**Table 1:** Quantitative comparisons on the two datasets.

Methods	ABIDE A^a^				ABIDE B^b^			
	ACC (%)	AUC (%)	SEN (%)	SPE (%)	ACC (%)	AUC (%)	SEN (%)	SPE (%)
Baseline-1	68.20 ± 5.13	73.02 ± 5.39	55.34 ± 10.86	79.30 ± 4.97	72.39 ± 3.04	81.92 ± 3.32	55.13 ± 6.30	86.04 ± 2.82
Baseline-2	78.30 ± 8.43	86.64 ± 7.71	75.45 ± 9.61	80.76 ± 9.43	80.92 ± 3.14	88.63 ± 1.96	77.81 ± 3.34	83.40 ± 5.52
ACERTA-ABIDE	64.07 ± 5.52	67.98 ± 7.32	40.52 ± 12.10	84.40 ± 2.69	82.41 ± 3.25	88.98 ± 2.65	75.19 ± 3.68	88.11 ± 4.31
ASD-DiagNet	67.74 ± 5.41	73.81 ± 5.47	62.79 ± 8.24	72.02 ± 5.33	85.04 ± 2.93	92.14 ± 1.95	81.63 ± 7.13	87.74 ± 3.07
population gcn	82.32 ± 7.33	89.34 ± 8.25	79.90 ± 10.80	84.39 ± 7.09	83.35 ± 3.79	91.32 ± 3.09	77.58 ± 8.17	87.92 ± 2.95
AIMAFE	61.53 ± 5.85	63.10 ± 6.93	34.94 ± 10.50	84.40 ± 4.70	81.04 ± 3.38	88.67 ± 3.58	73.75 ± 7.61	86.79 ± 3.16
MultiSurv	64.07 ± 5.92	67.82 ± 6.05	63.16 ± 18.50	64.98 ± 10.21	79.88 ± 5.04	91.23 ± 2.77	79.49 ± 12.73	80.19 ± 16.41
deepManReg	69.22 ± 6.25	77.97 ± 5.42	64.25 ± 11.06	73.52 ± 7.44	78.40 ± 1.88	85.85 ± 1.05	71.36 ± 5.08	83.96 ± 3.89
DeepASD	**87.38** ± **2.87**	**92.76** ± **4.00**	**88.35** ± **6.83**	**86.51** ± **8.41**	**88.09** ± **2.92**	**93.59** ± **2.45**	**87.58** ± **3.68**	**88.49** ± **4.73**

Best results are marked in bold.

^a^ABIDE A dataset consists of demographic information, automated anatomical quality assessment metrics, automated functional quality assessment metrics, and fMRI.

^b^ABIDE B dataset consists of fMRIs based on AAL, CC200, DOH atlas.

The shallow baselines include Baseline-1 [23], and Baseline-2 [24], which are commonly used in the biomedical field. The deep learning-based baselines are ACERTA-ABIDE [25], ASD-DiagNet [26], population-gcn [19], AIMAFE [10], MultiSurv [27], and deepManReg [28]. Baseline-1 [23] extracts the top 10 features of two modalities, such as clinical characteristics and laboratory test results, and then uses Random Forest to achieve higher accuracy. Baseline-2 [24] develops a predictive model using L1-regularised logistic regression (lasso) on multimodal features extracted from retinal fundus images, clinical measurements, and genomic data. In the ACERTA-ABIDE [25] study, Pearson correlation coefficients were computed for fMRI data, which were then fed into two stacked denoising autoencoders. This process was part of the unsupervised pre-training stage, aimed at extracting a lower-dimensional representation from the ABIDE dataset. Subsequently, the weights of these encoders were applied to a multilayer perceptron (MLP) for classification purposes. In the ASD-DiagNet [26] study, Pearson correlation calculations were performed on fMRI data. The approach included using features fused from the five nearest neighbors based on EROS similarity as a method for data augmentation. These features were subsequently input into an autoencoder, designed with tied weights, to extract a lower-dimensional feature representation. Finally, these reduced features were fed into a single-layer perceptron (SLP) for diagnostic classification. In the population-gcn [19] study, the construction of patient-patient relational graphs was based on demographic information, specifically sex and site. This structured graph then served as a framework into which fMRI data were input as features for classification through a graph neural network. AIMAFE [10] applies a stacked denoising autoencoder to learn multi-atlases deep feature representation and an ensemble learning method to conduct the ASD identification task. MultiSurv [27] integrates multiple feature extractors and multiple feature fusion methods to develop a deep learning model for cancer survival prediction. deepManReg [28] adopts multiple deep neural networks for different modalities, and jointly trains them to align multimodal features into a common latent space and then uses the cross-modal manifolds to regularize the classification network to improve phenotype predictions. Every model is able to take multimodal data as inputs by design.

### Qualitative performance of DeepASD

**Fig. 3:**
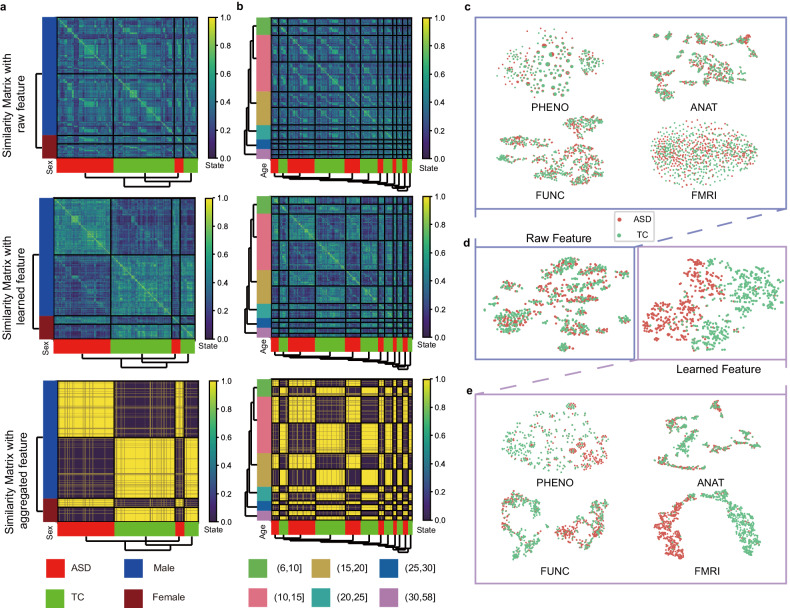
Visualization of cosine similarity heatmap across patients. The left column shows the results of clustering by sex (**a**), while the middle column is the results of clustering by age in the ABIDE A dataset (**b**). The first row represents the similarity matrices on raw features. The second row shows the similarity matrices of multimodal fused features after the multimodal adversarial-regularized encoder (details in Fig. 1b; Section “Multimodal adversarial-regularized encoder”. The last row presents the similarity matrices of fused features through GNN. There is a significant age and sex bias in the diagnosis of ASD, and the results show that the contrast between the diagnostics and the presentation of sex and age clusters, indicating that our multimodal adversarial-regularized encoder and multi-graph fusion GNN module are capable of learning more representative features for diagnosis. **c**–**e** are visualizations (t-SNE) of the feature representations in ABIDE A. In the three panels, the red color denotes ASD while green colors denotes TC. **c** Visualization of the raw features for each modality. **d** Visualization of raw features and learned features. The learned features are taken after the multimodal adversarial-regularized encoder. It’s clearly observed that the learned features are more distinguishable than the raw features. **e** Visualization of the learned features for each modality. Compared to the raw feature (**c**), the learned feature space (**e**) demonstrate that the fMRI has obvious class separability after feature extraction. In addition, the PHENO modality is also separable while the modalities of ANAT and FUNC are less divisible. The observation validates our explanation on the importance of modalities (Section “Multiple modalities enable more accurate diagnosis”; Fig. 4).

DeepASD learns a unified global graph where each node denotes a patient and the edge denotes the connections between patients (a.k.a. patient similarity networks). The node feature of the patient similarity network indicates patient representations learned from the multimodal data. Figure 3 visualizes the similarity matrix of patient representation to qualitatively evaluate the learned features. The heatmap shows the cosine similarity of different patient representations, indicating the features used for prediction have a high degree of sex and age specificity. From the first column of Fig. 3, we can see that the original features are not able to reveal the similarity related to sex and age, while the correlations between the learned patient similarity and demographic features (i.e., age and sex) tends to be clear by leveraging our proposed multimodal adversarial-regularized encoder and multi-graph fusion GNN module. The results demonstrate the bias of sex and age in the diagnosis process, which is consistent with the phenomenon described in (Wiggins, 2020) [20].

### The adversarial-regularized graph learning contributes to DeepASD

The proposed DeepASD mainly contains three components: the feature extractor, the adversarial modal discriminator, and the multi-graph fusion GNN. We first applied the feature extractor module to extract discriminative features from each modal, and then demonstrated the effectiveness of the remaining two components via an ablation study.

We used the ABIDE A data as an example for the ablation study. To verify the effectiveness of the multi-graph fusion GNN, we removed this module from DeepASD. As shown in Table 2, the AUC dropped from 92.76% to 82.99%, and there was an obvious decrease in terms of ACC, SEN, and SPE. Therefore, the learned patient similarity graphs from the multi-graph fusion GNN was able to improve the performance of ASD diagnosis. Similarly, the AUC dropped by 6.51% (from 82.99% to 77.59%) if the adversarial modal discriminator was removed. This is reasonable because the adversarial representation learning facilitates multi-modal feature alignment and eliminates the distribution gap between different modalities. Therefore, we can observe from the ablation study that the components in our DeepASD are effective to achieve a precise diagnosis of ASD.

**Table 2:** Ablation study.

Dataset	Modules			Metrics			
	Feature Extractor	Modal Discriminator	Multi-graph fusion GNN	ACC	AUC	SEN	SPE
ABIDE A	✓			72.78 ± 7.05	77.59 ± 3.97	64.35 ± 12.35	80.83 ± 9.94
			✓	72.22 ± 6.58	78.25 ± 6.06	65.79 ± 8.28	77.78 ± 0.07
	✓	✓		76.74 ± 3.79	82.99 ± 4.66	69.18 ± 12.36	83.38 ± 7.78
	✓		✓	83.47 ± 10.30	86.80 ± 10.14	80.12 ± 16.33	86.33 ± 11.03
	✓	✓	✓	**87.38** ± **2.87**	**92.76** ± **4.00**	**88.35** ± **6.83**	**86.51** ± **8.41**
ABIDE B	✓			86.05 ± 5.30	92.88 ± 3.26	81.72 ± 14.25	88.84 ± 3.94
			✓	86.29 ± 3.23	93.45 ± 1.70	82.21 ± 7.69	89.52 ± 3.66
	✓	✓		85.58 ± 4.29	91.78 ± 3.10	79.34 ± 8.07	**89.92** ± **2.25**
	✓		✓	86.51 ± 2.78	93.39 ± 2.38	83.53 ± 5.32	88.87 ± 4.12
	✓	✓	✓	**88.09** ± **2.92**	**93.59** ± **2.45**	**87.58** ± **3.68**	88.49 ± 4.73

Best results are marked in bold.

For a fair comparison, all the baselines use the same splits of the dataset to carry out a 10-fold stratified cross-validation strategy. Bold font indicates the best results. We adopt a 2-layer MLP as a feature extractor, which produces features that can be directly fed into the classifier for diagnosis. The combination of the feature extractor and modal discriminator constitute our multimodal adversarial-regularized encoder. On the ABIDE A dataset, the encoder outperforms the base MLP. By incorporating a multi-graph fusion GNN module, our DeepASD model is improved to achieve optimal results.

### Multiple modalities enable more accurate diagnosis

To assess the presence of confounding relationships between disease status and certain modalities of metadata, we first employed two-dimensional t-distributed stochastic neighbor embedding (t-SNE) [29] to visualize representations of each modality. It can be observed that there is no obvious clustering boundary of representations from raw features in the dataset (Fig. 3d left), while the representation generated by adversarial training of DeepASD is more discriminative, i.e., compact intra-class scatter and incompact inter-class scatter (Fig. 3d right). In addition, there is less overlapping across classes in each modality in Fig. 3c (top) than in Fig. 3e (bottom), indicating a better classification performance of the adversarial-regularized encoder in the DeepASD method. We also quantitatively compared the performance of DeepASD through the Silhouette score, and the score (0.5024) of DeepASD is much higher than that of in the raw data (0.4259), which validates the effectiveness of DeepASD in terms of representation learning.

**Fig. 4:**
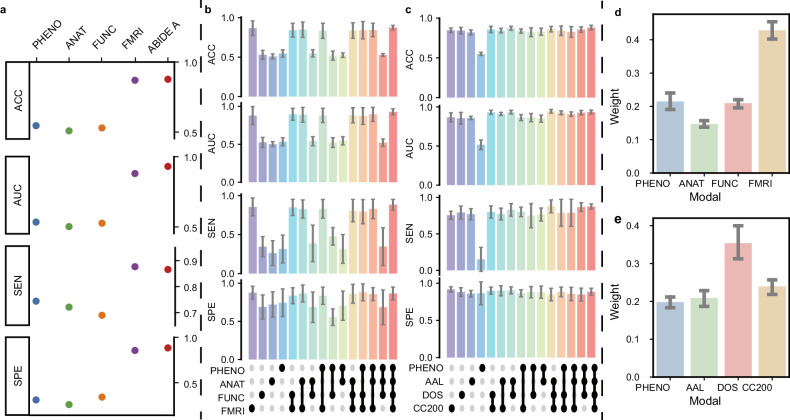
Visualization of modality importance. **a** The performance of training DeepASD on the multi-modal data and single-modality (PHENO, ANAT, FUNC, and fMRI, respectively). **b** Classification performance using different combinations of the four modalities (PHENO, ANAT, FUNC, FMRI) on the ABIDE A. The classifiers that contain the fMRI modality significantly outperform those without the fMRI modality, highlighting the significance of fMRI in the diagnostic process. Additionally, we also observe that combining auxiliary modalities with fMRI is able to further enhance its performance in ASD diagnosis. **c** Classification performance using different combinations of the four modalities (PHENO, AAL, DOS, CC200) on the ABIDE B. Though the classification results using the three brain atlas fMRI (AAL, DOS, CC200) are promising, the results are further improved by adding the PHENO modality data. **d**, **e** Illustration of the weight of each modality (on the ABIDE A and ABIDE B, respectively) in multi-graph fusion. The results show that fMRI is the most significant contributor in both datasets, which aligns with the results shown in **b** and **c**.

Due to the complexity of the mechanism of ASD, multimodal data are able to provide more comprehensive information for diagnosis. Here, we compared the performance of DeepASD using each modal separately as inputs in the ABIDE A such as demographic information (PHENO), automated anatomical quality assessment metrics (ANAT), automated functional quality assessment metrics (FUNC), and FMRI. Notably, the model achieved the best performance when using the FMRI modal alone, while the performance was further improved if the other three modalities are combined as the input (Fig. 4a, b). Interestingly, this is consistent with the importance of each modality learned by DeepASD model during the modal fusion using weighted multimodal feature representations (Fig. 4d).

We also conducted comparison experiments using each single modal and combinations of different modals in the ABIDE B as the inputs. It can be observed from Fig. 4c that DeepASD achieved a better performance for ASD diagnosis by combining the three brain atlases FMRI, and the DOS brain region is of importance for classifying ASD if only single modal was applied. Interestingly, it is also consistent with the learned importance of each modal in the fusion module (Fig. 4e) through DeepASD.

## Discussion

DeepASD aims to integrate multimodal clinical data for precise ASD diagnosis. The multimodal data were first projected into an intermediate common dimensional subspace through a multimodal adversarial-regularized encoder, which aligned the distributions of minor modalities with that of the anchor modality (i.e., fMRI). Importantly, DeepASD allows characterizing the latent patient similarity network for depicting inter-patient relationships, which facilitates the ASD diagnosis.

DeepASD benefits from the multimodal adversarial-regularized encoder and graph learning modules (Section “The adversarial-regularized graph learning contributes to DeepASD”), and achieved the best classification accuracy in terms of the diagnostic task compared to six benchmark models. DeepASD showed improved performance when trained with multiple modalities compared to single modality, indicating that fMRI is significant for ASD diagnosis, in which the DOS brain atlas is the most important fMRI modality.

Although DeepASD achieved superb performance, we noted that the performance of DeepASD may be decreased using low dimensional data. In the ABIDE A data, the dimensions of PHENO, ANAT, and FUNC are less than 50, while the dimension of fMRI is more than 100. From Fig. 3e, we observed that the separability of the low dimensional data became worse when projecting the low dimensional data (i.e., PHENO, ANAT, and FUNC) into the common dimension together with the high-dimensional fMRI data. Therefore, a key challenge of multi-modality fusion is how to exploit the information in low-dimensional clinical data.

We observed that DeepASD could be more effective by mimicking the psychiatric diagnostic process and integrating clinical autism rating scales [30, 31] as a modality. The autism scales are the gold standard in clinical diagnosis based on the performance of the clinical examination such as psychiatric examination, physical examination, laboratory tests, etc.

Interestingly, it should be noted that the interpretability of DeepASD indicated the importance of each modality, i.e., we observed that fMRI is the most significant modality for ASD diagnosis. However, one of the limitations of DeepASD is generalized to present more fine-grained interpretations. Therefore, the future work may include the interpretability in terms of the contribution of each feature for ASD diagnosis as in GradCAM [32], Shapley values [33], and DeepLIFT [34].

## Methods

### DeepASD overview

DeepASD receives $N$ patients data and each patient is associated with $K$ modalities. Let ${\bf{X}}=\{{{\bf{x}}}_{i}{\}}_{i=1}^{N}$ denotes the raw multimodal features of $N$ patients, and ${\bf{Y}}=\{{{\bf{y}}}_{i}{\}}_{i=1}^{N}$ denotes the corresponding labels of each patient. For patient $i$, the feature ${{\bf{x}}}_{i}=\{{{\bf{x}}}_{i}^{{m}_{1}},{{\bf{x}}}_{i}^{{m}_{2}},\cdots ,{{\bf{x}}}_{i}^{{m}_{k}}\}$ is composed of $K$ modalities, and we denote the modality by superscript ${m}_{k}$. For example, ${{\bf{x}}}_{i}^{{m}_{k}}$ denotes the ${d}_{{m}_{k}}$-dimensional features of $k$-th modality of the $i$-th patient. As shown in Fig. 1, for each modality, we fed ${{\bf{x}}}^{{m}_{k}}$ into feature extractor ${f}_{{m}_{k}}\left(\cdot \right)$ as input (Section “Feature extractor”). The ${f}_{{m}_{k}}\left(\cdot \right)$ generates aligned feature representation in ${{\mathbb{R}}}^{N\times {d}_{c}}$ through the multimodal adversarial-regularized encoder (Section “Multimodal adversarial-regularized encoder”), where ${d}_{c}$ represents the dimension of the aligned subspace. Then, in the multi-graph fusion GNN module (Section “Graph neural networks for multi-graph fusion”), we aggregate adjacency matrix $\{A{\}}_{k=1}^{K}$ and node embeddings $f\left(\cdot \right)=\{{f}_{{m}_{k}}\left({{\bf{x}}}^{{m}_{k}}\right){\}}_{k=1}^{K}$ in the patient similarity network (generated by all modalities), in order to generate one fusion adjacency matrix ${\mathscr{A}}{\mathscr{\in }}{{\mathbb{R}}}^{N\times N}$. Thus, a patient network $G=\left(V,E,{\bf{X}}\right)$ is built for ASD diagnosis, in which nodes represent patients $V$ and ${{\mathscr{A}}}_{{ij}}{\mathscr{\in }}{\mathscr{A}}$ denotes the edge weights of ${e}_{{ij}}\in E$. From the network, we employ Simple Spectral Graph Convolution (${\text{S}}^{2}$GC) [35] and a one-layer Multilayer Perceptron (MLP) to predict labels of nodes (i.e., the ASD prediction result of each patient).

### Feature extractor

Since each modality has its specific characteristics and patterns, we design a modality-specific extractor for each modal that feds each modal data into the module and projects the samples in each modality into a ${d}_{c}$-dimensional subspace, where we align the dimension of different modalities. Each feature extractor for each modal $\{{f}_{{m}_{k}}(\cdot )\!\!:{{\mathbb{R}}}^{{d}_{{m}_{k}}}\to {{\mathbb{R}}}^{{d}_{c}}\}_{k=1}^{K}$ is consisted of two-layer fully connected networks with the Leaky ReLU activation function, following with a one-layer fully connected networks for classification.

### Multimodal adversarial-regularized encoder

Adversarial networks [36, 37] have demonstrated effectiveness to align different data distributions. Since the features are heterogeneous across the modalities and each modality provides distinct information in terms of other modalities, we develop a multimodal adversarial-regularized encoder method to eliminate the feature heterogeneity and reduce the distributional divergence. As shown in Fig. 1b, we construct two competitive modules: the modal discriminator and the feature extractor. The modal discriminator $d\left(\cdot \right)$ aims to distinguish the modality of features, while the feature extractor $f\left(\cdot \right)$ attempts to against the former. By leveraging the adversarial learning manner, we are able to obtain aligned distributions from all modalities through training a competitive loss ${{\mathscr{L}}}_{d,f}$ (Eq. (1)) that minimizes over $d\left(\cdot \right)$ but maximizes over $f\left(\cdot \right)$.

1 $${{\mathscr{L}}}_{d,f}=\mathop{\sum }\limits_{k=1}^{K}\frac{1}{N}\mathop{\sum }\limits_{i=1}^{N}{L}_{s}\left[d({f}_{{m}_{k}}\left({{\boldsymbol{x}}}_{i}^{{m}_{k}}\right),{{\boldsymbol{z}}}_{i}^{{m}_{k}}\right].$$

where ${L}_{s}\left[\cdot ,\cdot \right]$ is the squared loss, and ${{\bf{z}}}_{i}^{{m}_{k}}$ is the one-hot labels of ${{\bf{x}}}_{i}^{{m}_{k}}$. In addition, there is a classification loss as shown in Eq. (2).

2 $${{\boldsymbol{L}}}_{f}=\mathop{\sum }\limits_{k=1}^{K}\frac{1}{N}\mathop{\sum }\limits_{i=1}^{N}{L}_{c}\left[f\left({{\boldsymbol{x}}}_{i}^{{m}_{k}}\right),{{\boldsymbol{y}}}_{i}\right]+\tau \left(\parallel f{\parallel }^{2}\right)$$

where ${L}_{c}\left[\cdot ,\cdot \right]$ is the cross-entropy loss and $\tau$ is a positive regularization parameter. The model tends to generate better discriminability via the feature extractor $f\left(\cdot \right)$ by minimizing ${{\mathscr{L}}}_{f}$. By combining two losses together, we have

3 $$\mathop{\min }\limits_{f}\mathop{\max }\limits_{d}{{\mathscr{L}}}_{f}-\beta {{\mathscr{L}}}_{d,f}$$

where $\beta$ is the trade-off parameter between the classification loss and the modal discriminator loss.

To alleviate the problem that the adversarial optimization on the ${{\mathscr{L}}}_{d,f}$ term may lead to vanishing gradients if $f\left(\cdot \right)$ and $g\left(\cdot \right)$ are not well synchronized, we adopt the invert label loss [38] as defined in Eq. (4):

4 $${\hat{{\mathscr{L}}}}_{d,f}=\mathop{\sum }\limits_{k=1}^{K}\frac{1}{N}\mathop{\sum }\limits_{i=1}^{N}{L}_{s}\left[d({f}_{{m}_{k}}\left({{\boldsymbol{x}}}_{i}^{{m}_{k}}\right),{\hat{{\boldsymbol{z}}}}_{i}^{{m}_{k}}\right].$$

where ${\hat{{\bf{z}}}}_{i}^{{m}_{k}}$ is the one-hot inverted label of ${{\bf{x}}}_{i}^{{m}_{k}}$ in each modal. Thus, the objective in Eq. (5) can be reformulated as

5 $$\mathop{\min }\limits_{f}{{\mathscr{L}}}_{f}+\beta {\hat{{\mathscr{L}}}}_{d,f},\mathop{\min }\limits_{d}{{\mathscr{L}}}_{d,f}$$

### Graph neural networks for multi-graph fusion

For multimodal features learned from the multimodal adversarial-regularized encoder, we apply a learnable cosine similarity [39] method in Eq. (6) to learn an inductive patient similarity graph, as follows,

6 $${A}_{{ij}}=\frac{{\left({W}_{A}{f}_{i}\right)}^{T}{W}_{A}{f}_{j}}{\parallel {W}_{A}{f}_{i}\parallel \parallel {W}_{A}{f}_{j}\parallel }$$

where ${A}_{{ij}}$ is the learned similarity matrix between patient $i$ and $j$, ${W}_{A}$ is the learned node embedding, ${f}_{i}$ and ${f}_{j}$ are the features from feature extractor $\{{f}_{{m}_{k}}\left({{\bf{x}}}_{i}^{{m}_{k}}\right){\}}_{k=1}^{K}$. We also employ a threshold $\theta$ to constrain the similarity strength between each node.

For each modality, we learn an adjacency matrix $\{A{\}}_{k=1}^{K}$ to capture patient relationships (a.k.a., similarity) in different modality, and then we combine all patient graphs into one graph whose adjacency matrix is ${\mathscr{A}}$ by weighted sum operation such that ${\mathscr{A}}{\mathscr{=}}\mathop{\sum }\limits_{k=1}^{K}{w}_{k}{A}_{k}.$ We obtain fused feature representations $\hat{f}$ by concatenating the aligned features from $\{{f}_{{m}_{k}}\left({{\bf{x}}}_{i}^{{m}_{k}}\right){\}}_{k=1}^{K}$. Based on the learned graph structure ${\mathscr{A}}{\mathscr{\in }}{{\mathbb{R}}}^{N\times N}$ and fusion feature $\hat{f}\in {{\mathbb{R}}}^{{d}_{c}}$, we apply ${\text{S}}^{2}$GC [35] and one-layer MLP for downstream ASD diagnosis task.

A spectral convolution of a graph signal $x$ with a filter ${g}_{\theta }$ is defined as ${g}_{\theta }\star x=U{g}_{\theta }{U}^{T}x$ where $U$ is the matrix of eigenvectors of the normalized graph Laplacian $L=\text{I}-{\text{D}}^{-\frac{1}{2}}{\mathscr{A}}{\text{D}}^{\frac{1}{2}}$ with respect to the diagonal degree matrix $D$. By the renormalization trick, we use a normalized version $\widetilde{{\rm{T}}}=\widetilde{{D}^{-\frac{1}{2}}}{\mathscr{A}}\widetilde{{D}^{\frac{1}{2}}}={\left(\text{D}+{\text{I}}_{\text{n}}\right)}^{-\frac{1}{2}}\left({\mathscr{A}}+{\text{I}}_{\text{n}}\right){\left(\text{D}+{\text{I}}_{\text{n}}\right)}^{-\frac{1}{2}}$ to replace the matrix $\text{I}-{\text{D}}^{-\frac{1}{2}}{\mathscr{A}}{\text{D}}^{\frac{1}{2}}$ Motivated by Markov Diffusion Kernel [40], ${\text{S}}^{2}$GC includes self-loops and its final output can be defined as follows,

7 $$\hat{Y}={softmax}\left(\frac{1}{C}\mathop{\sum }\limits_{c=1}^{C}\left(\left(1-\alpha \right){\widetilde{{\boldsymbol{T}}}}^{c}{\boldsymbol{X}}+\alpha {\boldsymbol{X}}\right){\boldsymbol{W}}\right)$$

where W represents the network parameter, and $\alpha$ is a trade-off between the self-information of a node and its consecutive neighborhoods. To this end, $\alpha$ is typically set to 0.05 with a range of values between 0 and 1 in the experiments. The term ${\widetilde{{\bf{T}}}}^{c}{\bf{X}}$ is computed as $\widetilde{{\bf{T}}}\cdot (\widetilde{{\bf{T}}}\cdot (\cdots (\widetilde{{\bf{T}}}{\bf{X}})\cdots ))$, where the multiplication is iteratively applied $C$ times.

Then, we build a final classifier to identify ASD. The classifier is composed of two-layer graph convolution layers with ReLU activation, followed by a fully connected layer. The loss function for this graph learning classification is given by Eq. (8)

8 $${{\mathscr{L}}}_{g}={L}_{c}\left[g\left({\mathscr{A}}{\mathscr{,}}\hat{f}\right),{\boldsymbol{y}}\right]$$

where ${L}_{c}\left[\cdot ,\cdot \right]$ is the cross-entropy loss, $g\left(\cdot ,\cdot \right)$ is the GCN model, ${\bf{y}}$ is the one-hot label of each patient data.

By combining all the aforementioned losses, we adopt the following joint loss function to guide the optimization of all modules simultaneously:

9 $${\mathscr{L}}{\mathscr{=}}{{\mathscr{L}}}_{g}+\eta \left({{\mathscr{L}}}_{f}+\beta {\hat{{\mathscr{L}}}}_{d,f}\right)$$

where $\eta$ and $\beta$ are hyper-parameters to balance the loss terms.

### Methodologies for training and testing

We first competitively train feature extractor $f\left(\cdot \right)$ and modal discriminator $d\left(\cdot \right)$ once, and then jointly train the graph construction embedding ${W}_{A}$ and GCN $g\left(\cdot ,\cdot \right)$ once in one training epoch. Through this training strategy, we are able to simultaneously obtain significant patient representations and patient similarity graphs with high prediction accuracy.

After selecting hyper-parameters through preliminary experiments, we optimize $f\left(\cdot \right)$, $d\left(\cdot \right)$, ${W}_{A}$, $g\left(\cdot ,\cdot \right)$ via the Adam optimizers [41] with learning rates of 0.004, 0.001, 0.001, 0.001, respectively. We empirically set $\beta =0.03$, $\tau =0.004$, and $\eta =1$, and the other hyper-parameters are fine-tuned according to the dataset size.

We implement the proposed DeepASD using PyTorch. All experiments were conducted with a 10-fold cross-validation to divide the dataset into training and test sets, with 10% of the training set randomly selected as the validation set. Ultimately, the training, validation, and test sets were non-overlapping. The training of the model for 500 epochs on ABIDE A and ABIDE B datasets, utilizing a single Tesla V100 GPU, took approximately 15 min and 45 min, respectively.

## Supplementary information

Supplementary File

Figure D1

## Data Availability

The ABIDE dataset is available from ref. [21], and the preprocessed dataset ABIDE A and ABIDE B are provided with the paper.

## Code availability

An interactive code base for DeepASD implementation is available at https://codeocean.com/capsule/6043886/tree.
