# Supplementary material for: DeepASD: a deep adversarial-regularized graph learning method for ASD diagnosis with multimodal data
Source: Transl Psychiatry. 2024 Sep 14;14:375. doi: 10.1038/s41398-024-02972-2 (PMC11401938; doi:10.1038/s41398-024-02972-2)
Supplement: Supplementary file 1 — Supplementary File [file 41398_2024_2972_MOESM1_ESM.docx]

# Appendix A Demographic information of the two datasets

The demographic information of the experimental subjects in two datasets is summarized in Table 1.

Table A1. Demographic information of the subjects in two datasets. The first column represents the 20 sites in the two datasets. TC/ASD denotes the number of negative/positive ASD samples in the datasets. Age denotes the mean and standard deviation of subjects. Male/Female denotes the number of objects with respect to the sex.

|  | | ABIDE A |  |  | ABIDE B |  |
| --- | --- | --- | --- | --- | --- | --- |
| Site | TC/ASD | Age | Male/Female | TC/ASD | Age | Male/Female |
| CALTECH | 10/5 | 26.79*±*10.40 | 10/5 | 18/14 | 27.36*±*10.46 | 25/7 |
| CMU | 5/6 | 26.82*±*4.59 | 7/4 | 13/13 | 26.69*±*5.66 | 20/6 |
| KKI | 21/12 | 10.31*±*1.26 | 24/9 | 28/18 | 10.03*±*1.24 | 34/12 |
| LEUVEN 1 | 14/14 | 22.43*±*3.45 | 28/0 | 15/9 | 22.38*±*3.16 | 24/0 |
| LEUVEN 2 | 16/12 | 14.17*±*1.45 | 21/7 | 19/11 | 14.17*±*1.35 | 22/8 |
| MAX MUN | 27/19 | 26.50*±*10.51 | 42/4 | 28/17 | 25.42*±*11.00 | 42/3 |
| NYU | 98/74 | 15.33*±*6.59 | 136/36 | 100/60 | 15.51*±*6.63 | 125/35 |
| OHSU | 13/12 | 10.81*±*1.72 | 25/0 | 14/9 | 10.87*±*1.76 | 23/0 |
| OLIN | 14/14 | 17.04*±*3.36 | 23/5 | 15/18 | 16.67*±*3.43 | 29/4 |
| PITT | 26/24 | 18.50*±*6.70 | 43/7 | 27/24 | 18.89*±*6.84 | 45/6 |
| SBL | 14/12 | 33.77*±*6.48 | 26/0 | 15/12 | 32.85*±*6.02 | 27/0 |
| SDSU | 19/8 | 14.36*±*1.89 | 21/6 | 22/9 | 14.32*±*1.85 | 24/7 |
| STANFORD | 13/12 | 9.99*±*1.60 | 18/7 | 20/17 | 9.89*±*1.57 | 30/7 |
| TRINITY | 25/19 | 17.03*±*3.46 | 44/0 | 25/19 | 16.90*±*3.45 | 44/0 |
| UCLA 1 | 27/37 | 13.35*±*2.36 | 55/9 | 31/33 | 13.07*±*2.29 | 55/9 |
| UCLA 2 | 10/11 | 12.47*±*1.63 | 19/2 | 13/13 | 12.49*±*1.50 | 24/2 |
| UM 1 | 52/34 | 13.77*±*2.94 | 61/25 | 53/46 | 13.44*±*2.93 | 75/24 |
| UM 2 | 21/13 | 16.01*±*3.31 | 32/2 | 21/12 | 16.05*±*3.35 | 31/2 |
| USM | 24/43 | 22.59*±*8.30 | 67/0 | 25/42 | 22.74*±*8.51 | 67/0 |
| YALE | 19/22 | 13.31*±*2.61 | 25/16 | 28/23 | 12.79*±*2.86 | 37/14 |
| total | 468/403 | 16.94*±*7.58 | 727/144 | 530/419 | 16.88*±*7.75 | 803/146 |

# Appendix B Classification Performance on the two datasets

The detailed results of the classification performance are shown in Table 2.

Table B2. Quantitative comparisons on the two datasets.

| Methods | ABIDE A^[[1]](#footnote-1)^ | | | | ABIDE B^[[2]](#footnote-2)^ | | | |
| --- | --- | --- | --- | --- | --- | --- | --- | --- |
|  | ACC (%) | AUC (%) | SEN (%) | SPE (%) | ACC (%) | AUC (%) | SEN (%) | SPE (%) |
| Baseline-1 | 68.20*±*5.13 | 73.02*±*5.39 | 55.34*±*10.86 | 79.30*±*4.97 | 72.39*±*3.04 | 81.92*±*3.32 | 55.13*±*6.30 | 86.04*±*2.82 |
| Baseline-2 | 78.30*±*8.43 | 86.64*±*7.71 | 75.45*±*9.61 | 80.76*±*9.43 | 80.92*±*3.14 | 88.63*±*1.96 | 77.81*±*3.34 | 83.40*±*5.52 |
| ACERTA-ABIDE | 64.07*±*5.52 | 67.98*±*7.32 | 40.52*±*12.10 | 84.40*±*2.69 | 82.41*±*3.25 | 88.98*±*2.65 | 75.19*±*3.68 | 88.11*±*4.31 |
| ASD-DiagNet | 67.74*±*5.41 | 73.81*±*5.47 | 62.79*±*8.24 | 72.02*±*5.33 | 85.04*±*2.93 | 92.14*±*1.95 | 81.63*±*7.13 | 87.74*±*3.07 |
| population gcn | 82.32*±*7.33 | 89.34*±*8.25 | 79.90*±*10.80 | 84.39*±*7.09 | 83.35*±*3.79 | 91.32*±*3.09 | 77.58*±*8.17 | 87.92*±*2.95 |
| AIMAFE | 61.53*±*5.85 | 63.10*±*6.93 | 34.94*±*10.50 | 84.40*±*4.70 | 81.04*±*3.38 | 88.67*±*3.58 | 73.75*±*7.61 | 86.79*±*3.16 |
| MultiSurv | 64.07*±*5.92 | 67.82*±*6.05 | 63.16*±*18.50 | 64.98*±*10.21 | 79.88*±*5.04 | 91.23*±*2.77 | 79.49*±*12.73 | 80.19*±*16.41 |
| deepManReg | 69.22*±*6.25 | 77.97*±*5.42 | 64.25*±*11.06 | 73.52*±*7.44 | 78.40*±*1.88 | 85.85*±*1.05 | 71.36*±*5.08 | 83.96*±*3.89 |
| DeepASD | **87.38***±***2.87** | **92.76***±***4.00** | **88.35***±***6.83** | **86.51***±***8.41** | **88.09***±***2.92** | **93.59***±***2.45** | **87.58***±***3.68** | **88.49***±***4.73** |

# Appendix C Ablation Study of Data Modality on the Classification Performance.

We validate DeepASD by various data modality combinations and report classification performance in Table 3 and [Table 4](#tab:modality_compare_ B). From Table 3, we can observe that data modalities with FMRI modality show higher accuracy, AUC, sensitivity, and specificity compared with using data only containing non-imaging data (PHENO, ANAT, FUNC). Therefore, we conclude that FMRI significantly contributes to ASD diagnosis, which is consistent with the importance of clinical application in real-world diagnosis. However, we are not able to distinguish their importance from the three modalities of AAL, CC200, and DOS in ABIDE B dataset, as shown in Table 4. The three modalities may have similar contribution to ASD diagnosis.

Table C3. Comparison of DeepASD on various data combinations on the ABIDE A. For a fair comparison, all the modality combinations use the same dataset splits to carry out a 10-fold stratified cross-validation strategy and adopt the same training strategy. Bold font indicates the best results.

| Data | ACC (%) | AUC (%) | SEN (%) | SPE (%) |
| --- | --- | --- | --- | --- |
| PHENO | 54.53*±*4.98 | 53.35*±*5.59 | 31.47*±*18.84 | 74.30*±*19.10 |
| ANAT | 50.86*±*3.37 | 50.15*±*3.54 | 26.32*±*16.76 | 71.98*±*17.60 |
| FUNC | 52.93*±*6.03 | 52.56*±*6.98 | 34.54*±*13.65 | 68.84*±*16.70 |
| FMRI | 86.68*±*9.72 | 87.72*±*12.56 | 85.57*±*12.20 | 87.60*±*8.81 |
| PHENO+ANAT | 52.46*±*3.07 | 54.63*±*5.74 | 31.59*±*19.50 | 70.56*±*20.20 |
| PHENO+FUNC | 51.90*±*6.12 | 52.30*±*6.79 | 47.71*±*11.89 | 55.55*±*11.52 |
| PHENO+FMRI | 83.34*±*10.13 | 88.03*±*9.94 | 83.08*±*12.60 | 83.52*±*12.00 |
| ANAT+FUNC | 54.76*±*4.83 | 54.03*±*6.37 | 38.75*±*24.80 | 68.53*±*21.05 |
| ANAT+FMRI | 84.84*±*10.24 | 88.79*±*10.31 | 82.82*±*12.37 | 86.51*±*11.76 |
| FUNC+FMRI | 84.15*±*9.28 | 89.46*±*8.34 | 84.90*±*11.02 | 83.57*±*10.98 |
| PHENO+ANAT+FUNC | 52.70*±*1.83 | 52.14*±*5.20 | 34.57*±*25.57 | 68.43*±*23.98 |
| PHENO+ANAT+FMRI | 84.38*±*8.98 | 89.43*±*9.75 | 82.83*±*13.11 | 85.68*±*8.93 |
| PHENO+FUNC+FMRI | 84.05*±*11.59 | 87.17*±*11.67 | 79.45*±*16.37 | **88.05***±***11.69** |
| ANAT+FUNC+FMRI | 83.69*±*10.37 | 88.18*±*10.51 | 80.87*±*15.12 | 86.10*±*13.07 |
| **PHENO+ANAT+FUNC+FMRI** | **87.38***±***2.87** | **92.76***±***4.00** | **88.35***±***6.83** | 86.51*±*8.41 |

PHENO: demographic information; ANAT: automated anatomical quality assessment metrics; FUNC: automated functional quality assessment metrics; FMRI: functional magnetic resonance imaging. All statistic information are mean and standard deviation.

Table C4. Comparison of DeepASD on various data combinations on the ABIDE B. For a fair comparison, all the modality combinations use the same dataset splits to carry out a 10-fold stratified cross-validation strategy and adopt the same training strategy. Bold font indicates the best results.

| Data | ACC (%) | AUC (%) | SEN (%) | SPE (%) |
| --- | --- | --- | --- | --- |
| PHENO | 54.90*±*2.10 | 51.67*±*6.41 | 15.08*±*17.41 | 86.42*±*16.20 |
| AAL | 81.98*±*3.43 | 85.90*±*2.34 | 77.10*±*8.08 | 85.85*±*4.73 |
| DOS | 84.29*±*4.28 | 85.50*±*7.94 | 79.43*±*9.37 | 88.11*±*6.04 |
| CC200 | 84.62*±*3.06 | 86.67*±*6.13 | 75.66*±*5.92 | **91.70***±***3.79** |
| PHENO+AAL | 82.93*±*4.72 | 85.00*±*4.80 | 76.60*±*9.42 | 87.92*±*8.68 |
| PHENO+DOS | 82.28*±*5.92 | 86.37*±*5.58 | 75.08*±*17.51 | 87.92*±*7.97 |
| PHENO+CC200 | 83.67*±*2.77 | 86.29*±*4.11 | 80.19*±*6.94 | 86.42*±*5.02 |
| AAL+DOS | 87.14*±*2.71 | 93.34*±*2.45 | 83.07*±*9.20 | 90.38*±*4.99 |
| AAL+CC200 | 84.30*±*3.03 | 91.11*±*2.01 | 77.35*±*8.53 | 89.81*±*7.13 |
| DOS+CC200 | 85.66*±*4.15 | 93.15*±*2.84 | 80.16*±*8.81 | 90.00*±*5.04 |
| PHENO+AAL+DOS | 85.67*±*3.69 | 92.72*±*3.06 | 86.89*±*6.36 | 84.72*±*9.09 |
| PHENO+AAL+CC200 | 82.61*±*7.14 | 90.83*±*3.06 | 79.02*±*19.71 | 85.47*±*9.58 |
| PHENO+DOS+CC200 | 84.19*±*6.30 | 92.53*±*2.61 | 78.99*±*19.51 | 88.30*±*6.88 |
| AAL+DOS+CC200 | 86.09*±*3.74 | **94.36***±***2.52** | 87.83*±*9.35 | 84.72*±*8.96 |
| **PHENO+AAL+DOS+CC200** | **88.09***±***2.92** | 93.59*±*2.45 | **87.58***±***3.68** | 88.49*±*4.73 |

AAL: AAL atlas-based FC; DOS: Dosenbach160 atlas-based FC; CC200: CC200 atlas-based FC. All statistic information are mean and standard deviation.

# Appendix D Visualization of the learned features on the ABIDE B

In Fig. D1a and Fig. D1b, we visualize the similarity matrix of patient representations to qualitatively evaluate the learned features on the ABIDE B dataset. In Fig. D1c, Fig. D1d and Fig. D1e, we use two-dimensional t-distributed stochastic neighbor embedding (t-SNE) to visualize the low-dimensional representations of patient based on the multimodal data.

# Appendix E Classification Performance on the non-clinical dataset.

We first conducted an experiment to validate our DeepASD model on the Pascal VOC dataset, which includes 9963 image-text pairs with 20 classes. In our experiment, we selected 5649 pairs where the image contains only one object. Each image in the dataset is represented by a 512-dimensional feature vector, and each text is represented by a 399-dimensional word frequency count vector. Table 5. Classification Performance on the Pascal VOC dataset.presents the classification performance of DeepASD and other state-of-the-art deep learning-based methods, including ACERTA-ABIDE, ASD-DiagNet, population_gcn, AIMAFE, MultiSurv, and deepManReg, as well as traditional machine learning-based methods Baseline-1 and Baseline-2, on the Pascal VOC dataset in terms of accuracy (ACC) and area under the curve (AUC). The table demonstrates that the DeepASD achieved superior overall performance when considering all the evaluation metrics. Comparing with baseline methods, DeepASD outperforms most of the baselines (i.e., MultiSurv, deepManReg, and Baseline-1) in terms of accuracy and AUC while performs slightly worse than AIMAFE and Baseline-2 in terms of AUC. Specifically, compared with other methods, DeepASD achieves higher accuracy and AUC, demonstrating its potential in various classification tasks.

Table E5. Classification Performance on the Pascal VOC dataset.

| Method | ACC (%) | AUC (%) |
| --- | --- | --- |
| Baseline-1 | 85.55*±*0.98 | 96.29*±*1.37 |
| Baseline-2 | 85.06*±*0.98 | 98.26*±*0.93 |
| ACERTA-ABIDE | 83.27*±*0.84 | 95.61*±*0.83 |
| ASD-DiagNet | 77.96*±*1.33 | 96.58*±*1.23 |
| population gcn | 61.51*±*7.76 | 78.35*±*3.69 |
| AIMAFE | 67.62*±*1.82 | 98.69*±*0.45 |
| MultiSurv | 85.33*±*1.89 | 95.49*±*1.31 |
| deepManReg | 79.36*±*1.83 | 89.36*±*1.17 |
| DeepASD | 90.83*±*1.04 | 96.90*±*0.78 |

# Figures

**Fig. D1.** Visualization of the learned feature on the ABIDE B. **a** and **b** visualize cosine similarity across patients on ABIDE B. The first column is the result of clustering in terms of sex on the ABIDE B dataset (**a**), and the middle column is the result of clustering in terms of age (**b**). The top row represents the similarity matrices of raw features. The second row shows the similarity matrices of multimodal fusion features after the multimodal adversarial-regularized encoder (details in Figure [1](#framework) **c**). The last row presents the similarity matrices of fused features through GCN. We can find that the contrast between the similarity matrix of features from left to right by category and the sex and age clusters is getting higher, indicating that our multimodal adversarial-regularized encoder and multi-graph fusion GNN module can learn more representative features for diagnosis. **c**, **d** and **e** are visualization (t-SNE) of the feature representations in ABIDE B. The red color denotes ASD and green color denotes TC. **c** Visualization of raw features in each modality. **d** Visualization of raw features and learned features. **e** Visualization of learned features of each modality after the graph convolution layer that are final features to be fed into the ASD classifier. We observe that the learned features from our multimodal adversarial-regularized encoder and multi-graph fusion GNN module are more discriminative than using the raw features.

1. ABIDE A dataset consists of demographic information, automated anatomical quality assessment metrics, automated functional quality assessment metrics, and fMRI. [↑](#footnote-ref-1)
2. ABIDE B dataset consists of fMRIs based on AAL, CC200, DOH atlas. [↑](#footnote-ref-2)
