# Supplementary figures and images for: DeepASD: a deep adversarial-regularized graph learning method for ASD diagnosis with multimodal data
Source: Transl Psychiatry. 2024 Sep 14;14:375. doi: 10.1038/s41398-024-02972-2 (PMC11401938; doi:10.1038/s41398-024-02972-2)

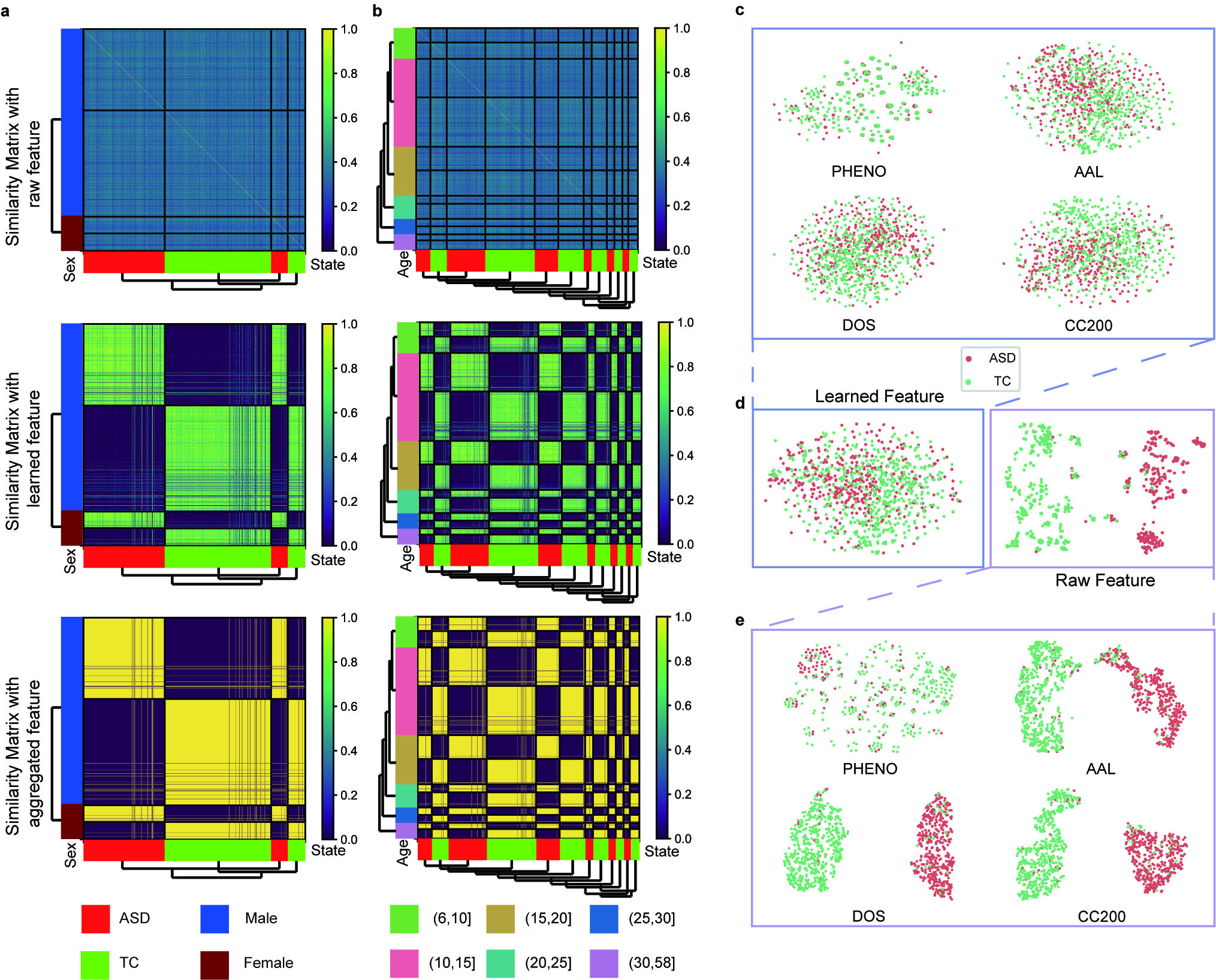

Supplement: Supplementary file 2 — Figure D1 [file 41398_2024_2972_MOESM2_ESM.tif]
